# Supplementary material for: Novel Driver Strength Index highlights important cancer genes in TCGA PanCanAtlas patients
Source: PeerJ. 2022 Aug 11;10:e13860. doi: 10.7717/peerj.13860 (PMC9375969; doi:10.7717/peerj.13860)
Supplement: Supplemental Information 2 [file peerj-10-13860-s002.zip › COHORTS/genes plots/age/2021_8_16_14_9_distribution_cohorts_males_25-29.pdf]

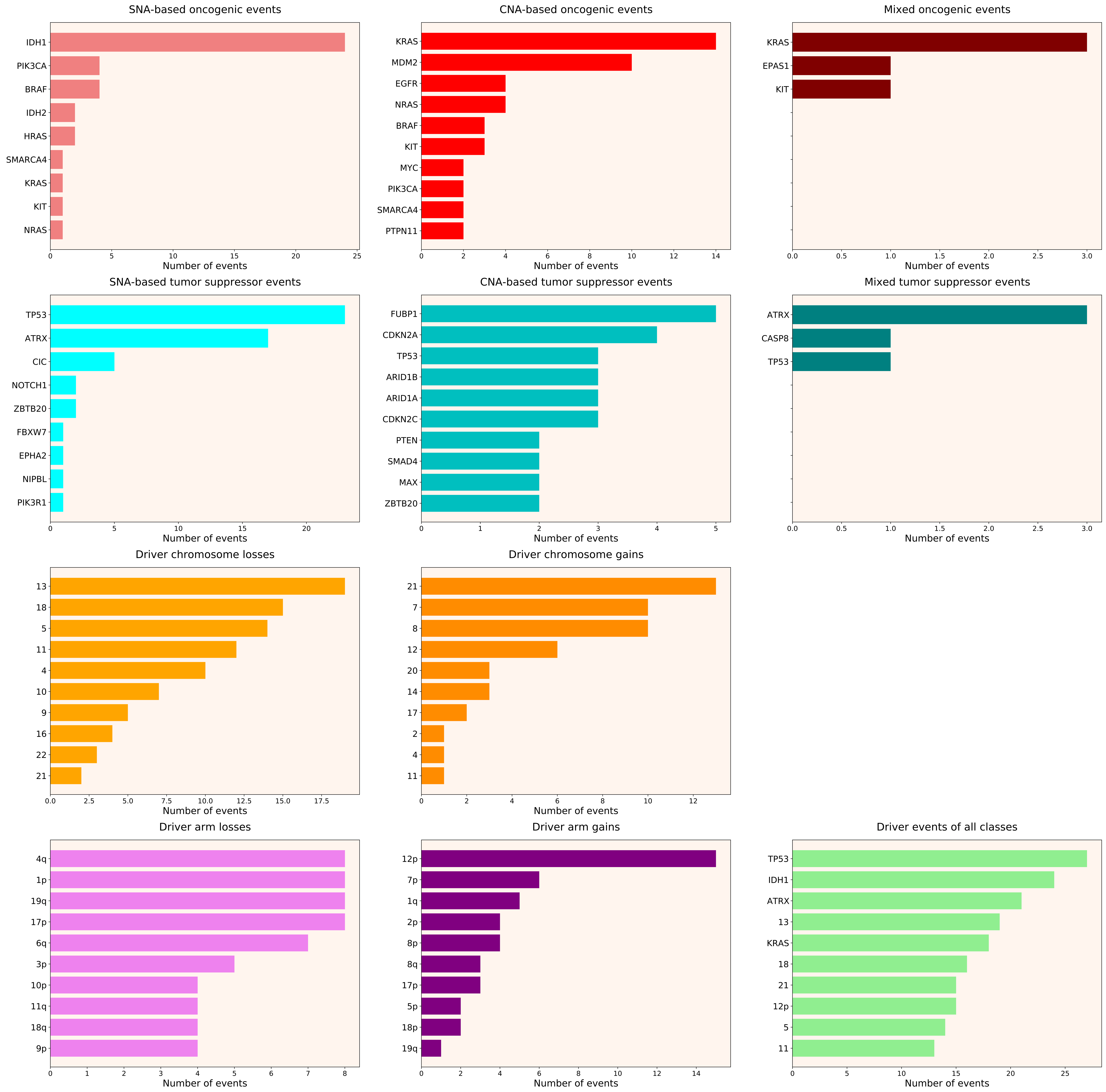

SNA-based oncogenic events
IDH1
PIK3CA
BRAF
IDH2
HRAS
SMARCA4
KRAS
KIT
NRAS
0 5 10 15 20 25
Number of events
CNA-based oncogenic events
KRAS
MDM2
EGFR
NRAS
BRAF
KIT
MYC
PIK3CA
SMARCA4
PTPN11
0 2 4 6 8 10 12 14
Number of events
Mixed oncogenic events
KRAS
EPAS1
KIT
0.0 0.5 1.0 1.5 2.0 2.5 3.0
Number of events
SNA-based tumor suppressor events
TP53
ATRX
CIC
NOTCH1
ZBTB20
FBXW7
EPHA2
NIPBL
PIK3R1
0 5 10 15 20
Number of events
CNA-based tumor suppressor events
FUBP1
CDKN2A
TP53
ARID1B
ARID1A
CDKN2C
PTEN
SMAD4
MAX
ZBTB20
0 1 2 3 4 5
Number of events
Mixed tumor suppressor events
ATRX
CASP8
TP53
0.0 0.5 1.0 1.5 2.0 2.5 3.0
Number of events
Driver chromosome losses
13
18
5
11
4
10
9
16
22
21
0.0 2.5 5.0 7.5 10.0 12.5 15.0 17.5
Number of events
Driver chromosome gains
21
7
8
12
20
14
17
2
4
11
0 2 4 6 8 10 12
Number of events
Driver arm losses
4q
1p
19q
17p
6q
3p
10p
11q
18q
9p
0 1 2 3 4 5 6 7 8
Number of events
Driver arm gains
12p
7p
1q
2p
8p
8q
17p
5p
18p
19q
0 2 4 6 8 10 12 14
Number of events
Driver events of all classes
TP53
IDH1
ATRX
13
KRAS
18
21
12p
5
11
0 5 10 15 20 25
Number of events
